# Bisphenol A Impairs Synaptic Plasticity by Both Pre‐ and Postsynaptic Mechanisms

**DOI:** 10.1002/advs.201600493

**Published:** 2017-04-19

**Authors:** Fan Hu, Tingting Li, Huarui Gong, Zhi Chen, Yan Jin, Guangwei Xu, Ming Wang

**Affiliations:** ^1^ School of Food Science and Engineering Hefei University of Technology Hefei Anhui 230009 P. R. China; ^2^ CAS Key Laboratory of Brain Function and Diseases School of Life Sciences University of Science and Technology of China Hefei Anhui 230027 P. R. China

**Keywords:** bisphenol A, hippocampus, spatial memory, spine, synaptic plasticity

## Abstract

Bisphenol A (BPA), an environmental xenoestrogen, has been reported to induce learning and memory impairments in rodent animals. However, effects of BPA exposure on synaptic plasticity and the underlying physiological mechanisms remain elusive. Our behavioral and electrophysiological analyses show that BPA obviously perturbs hippocampal spatial memory of juvenile Sprague–Dawley rats after four weeks exposure, with significantly impaired long‐term potentiation (LTP) in the hippocampus. These effects involve decreased spine density of pyramidal neurons, especially the apical dendritic spine. Further presynaptic findings show an overt inhibition of pulse‐paired facilitation during electrophysiological recording, which suggest the decrease of presynaptic transmitter release and is consistent with reduced production of presynaptic glutamate after BPA exposure. Meanwhile, LTP‐related glutamate receptors, NMDA receptor 2A (NR2A) and AMPA receptor 1 (GluR1), are significantly downregulated in BPA‐exposed rats. Excitatory postsynaptic currents (EPSCs) results also show that EPSC_NMDA_, but not EPSC_AMPA_, is declined by 40% compared to the baseline in BPA‐perfused brain slices. Taken together, these findings reveal that juvenile BPA exposure has negative effects on synaptic plasticity, which result from decreases in dendritic spine density and excitatory synaptic transmission. Importantly, this study also provides new insights into the dynamics of BPA‐induced memory deterioration during the whole life of rats.

## 1 Introduction

Bisphenol A (BPA) is a synthetic compound widely used in producing plastics and epoxy resins. Every year, there is more than 6 billion pounds demanding of BPA for producing dental sealants, thermal receipts, food packaging, plastics bottles, and so on.^1^ It has been found a broad distribution of BPA in environmental milieus including food, water, dust, and soil.^2^ A large body of evidence reveals that BPA exposure is related to adverse health effects, e.g., low‐birth weight, reproductive problem, obesity, diabetes, cardiovascular diseases, and cancers.^3^

As a xenoestrogen, BPA could exert hormone mimetic or antagonist activities through binding estrogen receptor α and β.^4^ BPA could cross the blood–brain barrier (BBB)^5^ and then alter neuronal behaviors in different life stages and species. For example, chronic BPA exposure in pregnancy and lactation (the critical periods of brain development) could induce memory deficits later in the life of rats, such as impaired spatial recognition memory in mid‐adolescence,^6^ and deficient spatial learning and memory in juvenile and adult.^7^ In adolescent rats (49–60 d old), BPA exposure impairs spatial memory in the object placement test, which is independent of the gender.^8^ Specifically, in monkeys, working memory was also decreased after four weeks BPA exposure.[[qv: 5b]] These memory changes in rodents and primates are usually accompanied by loss of dendritic spines in the medial prefrontal cortex and hippocampus,[[qv: 5b,9]] which provide possible morphological mechanisms behind learning and memory deficits in BPA‐exposed animals. To fully understand the mechanisms underlying the declined cognitive behaviors, it is necessary to examine synaptic plasticity of related cortices at the physiological level. Currently, reports assessing how physiological functions degrade in BPA‐exposed rodents are scarce.

It is well known that the demand for synaptic plasticity keeps altering in the course of a lifetime.^10^ Exploring different stages of BPA‐induced function deterioration would shed valuable light on the causal links among various aspects of synaptic impairment and, eventually, provide more information on how BPA affects cognitive behaviors. According to previous studies, BPA exposure times are mostly gestation and lactation in rodents or primates. Although this phase is considered as critical brain developmental window with high synaptic plasticity,^11^ the juvenile period is also important with brain in its final development phase and the gray matter of the frontal and parietal lobes peaks at about age 12 (the end of the juvenile period) in humans.^12^ The current study aims to assess whether juvenile BPA exposure perturbs hippocampus‐dependent learning and memory in rats; exploring the underlying mechanism by evaluating synaptic plasticity (e.g., long‐term plasticity), dendritic morphology, and synaptic transmission. Our findings provide important physiological evidence for cognitive deficit, and a crucial “missing link” between the pre‐ and postjuvenile phases of BPA‐induced functional deterioration.

## 2 Results

### 2.1 BPA Penetrated the Blood–Brain Barrier

**Figure 1:**
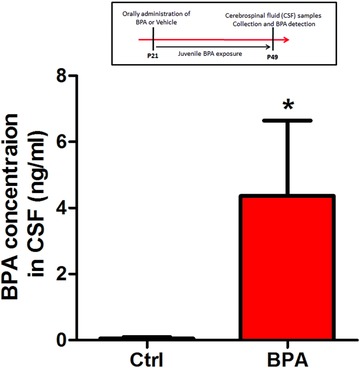
BPA crosses the BBB in CNS. Cerebrospinal fluid (CSF) samples were collected from the third ventricle of rats with or without juvenile BPA exposure at P49. BPA levels in CSF were determined by UPLC. Histograms were plotted by the mean of eight rat hippocampus per group. (*p < 0.05, *n* = 8 rats per group).

To assess the effect of juvenile BPA exposure on behavioral function, we first examined whether BPA could penetrate the blood–brain barrier (BBB) in CNS. BPA concentrations in cerebrospinal fluid (CSF) samples from the third ventricle were determined by ultra‐performance liquid chromatography (UPLC). The retention time of BPA in column was 3.66 min and the standard curves showed a linear range at 0.2–25 ng mL^−1^ (*y* = 6.8 × 10^−5^ *x*, *r* ^2^ = 0.99). As shown in **Figure** **1**, exposure of juvenile rats for 38 d to BPA resulted in a mean CSF level of 4.36 ± 2.28 ng mL^−1^; BPA concentration in control group was 0.17 ± 0.07 ng mL^−1^ (*n* = 8 rats per group, *p* < 0.05).

### 2.2 BPA Exposure Impairs Hippocampus‐Dependent Spatial Memory

**Figure 2:**
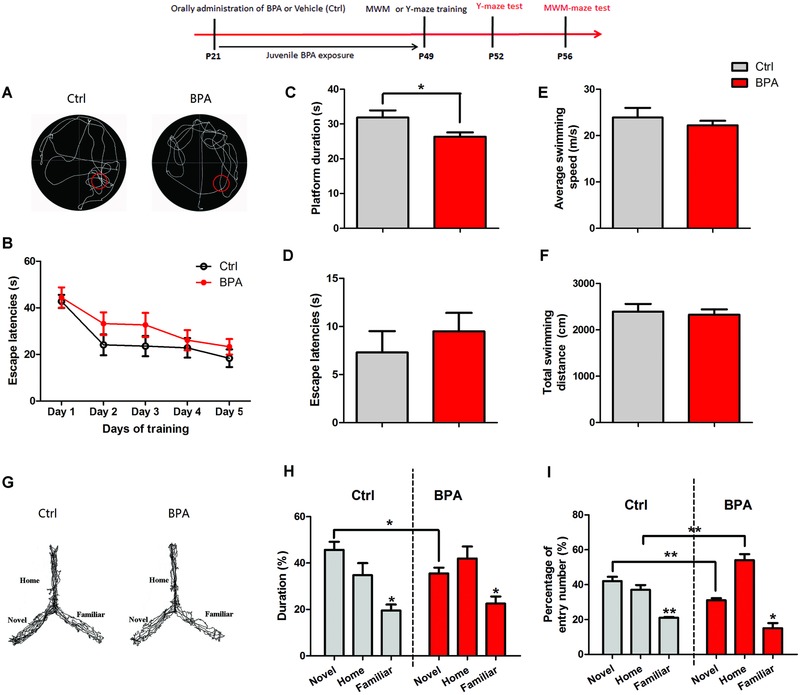
Deterioration of spatial memory by juvenile BPA exposure. A) Rats after chronic oral treatment of BPA (*n* = 8 rats, red) or DMSO (*n* = 8 rats, black) were assessed for spatial memory by the Marris‐water maze test, as shown by B) escape latency to reach the platform after 5 d of training (*p* > 0.05), C) platform duration (*p < 0.05), D) escape latency (*p* > 0.05), and E,F) swimming speed and distance (*p* > 0.05). G) Rats (*n* = 8 rats per group) were assessed for spatial memory in the Y maze test (without stress punishment or reward), as shown by H) the percentage of duration (*p < 0.05) and I) entry number in the novel arm (**p < 0.01), and I) percentage of entry number in the home arm (**p < 0.01).

Next, we addressed whether juvenile BPA exposure results in the deterioration of spatial learning and memory. We first conducted the classic Morris Water Maze test in juvenile rats after chronic BPA exposure from weaning (P21) to 49 d old (P49) (*n* = 8 rats per group). During training (four times a day, at 10 min intervals), there is no significant difference on the time for finding the escape platform between control and BPA‐exposed rats (*p* > 0.05, **Figure** **2**B). In probe tests performed at the end of training, the platform was removed and the rats were allowed to search for 90 s. As shown in Figure 2C, BPA‐treated rats spent significantly less swimming time in the target quadrant (location of platform) than the control animals (*p* < 0.05). During probe test, escape latency of BPA‐treated rats showed no significant difference with the control group, but an increasing trend (*p* > 0.05, Figure 2D). Increased escape time would cause the decline of duration in the target quadrant. The current training and probe test results suggest that juvenile BPA exposure resulted in hippocampal‐dependent spatial memory impairment without learning deficit. Notably, BPA‐treated rats showed no difference in swimming speed and total swimming distance with control rats (*p* > 0.05, Figure 2E,F).

Because the Morris‐water maze (MWM) test represents an artificial situation and is relatively stressful for rats, a less stressful and ethologically more relevant spatial‐memory assay, the Y maze test, was also used (*n* = 8 rats per group). As shown in Figure 2G, although all rats had a preference for entering into the novel arm (previously unvisited arm) of the maze for higher duration and entry number than the familiar arm (*p* < 0.05, Figure 2H,I), the BPA group spent less time in the novel arm than the control group (*p* < 0.05, Figure 2H). Meanwhile, BPA‐exposed rats had lower frequency of entry into the novel arm and higher frequency of entry into the home arm than the control group (*p* < 0.01, Figure 2I). These findings suggest the deficits of spatial memory for familiar environment in juvenile‐BPA‐exposed rats. Additionally, the locomotion of rats (moving distance and velocity) in open field test was similar between the two groups (Supporting Information), which strongly implicated that memory deficits were not due to motor dysfunction.

### 2.3 BPA Exposure Impairs Basic Synaptic Transmission and Long‐Term Plasticity of Sch‐CA1 Pathway in Hippocampus

**Figure 3:**
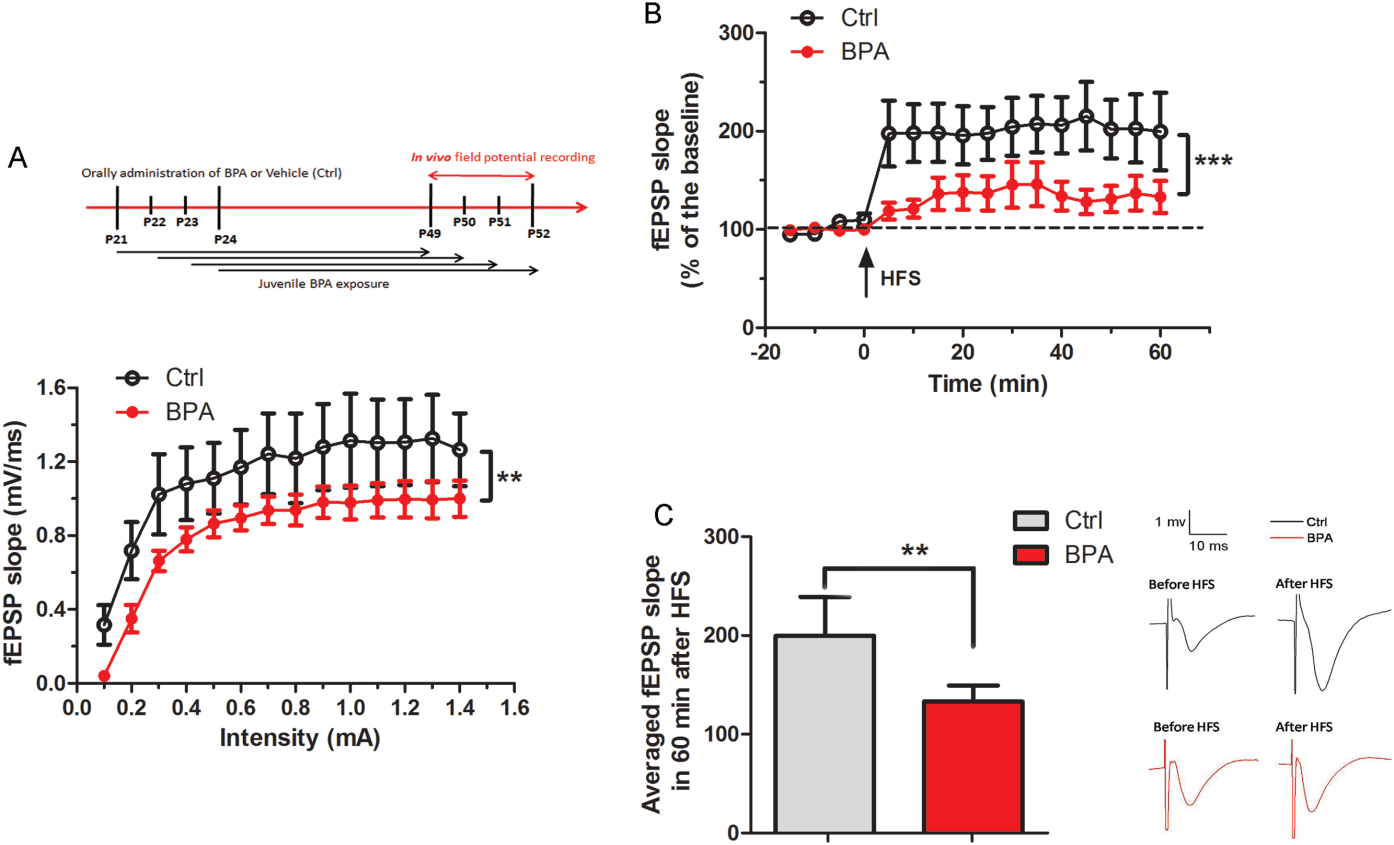
Impairment of basic synaptic transmission and long‐term plasticity (LTP) in the hippocampal CA1 areas after juvenile BPA exposure. A) Input/output (*I*/*O*) curves showed the fEPSP slopes as a function of stimulus current intensities in control and BPA‐exposed groups. *I*/*O* was significantly depressed in BPA‐exposed rats compared with that of the control value (*n* = 10 rats per group, **p < 0.01). B) The magnitude of LTP was assessed by the fEPSP slope (percentage of baseline) in every 5 min within 60 min after high frequency tetanic stimulation (HFS) (*n* = 10 rats per group, ***p < 0.001). C) Histogram showing a significant decrease of LTP magnitude in 60 min of juvenile BPA‐exposed rats (*n* = 10 rats per group, **p < 0.01). Arrow indicates of HFS application.

Before exploring synaptic plasticity, basic synaptic potency was analyzed by measuring the conductivity of Schaffer collateral (Sch)‐CA1 synapse. Compared to the control group, the *I*/*O* curve of field excitatory postsynaptic potential (fEPSP) slope in BPA exposure group was significantly depressed with a range of stimulus intensities (0.1–1.4 mA) (*n* = 10 rats per group, *p* < 0.01, **Figure** **3**A). This result suggests that juvenile BPA exposure impaired the basic synaptic transmission of the hippocampal CA1 areas.

Synaptic plasticity, including long‐term potentiation and depression (LTP and LTD), has been recognized as the cellular basis of learning and memory at the physiological level. Therefore, we assessed LTP induction in the hippocampal CA1 areas after high‐frequency stimulation (HFS) of Sch (Figure 3B). As shown in Figure 3C, BPA exposure produced a 133.11 ± 16.17% potentiation of fEPSP slope, whereas control LTP was 199.63 ± 39.51% of fEPSP in 60 min after HFS (*p* < 0.01). The normalized results indicate that BPA exposure significantly impaired synaptic plasticity of the hippocampal CA1 areas.

### 2.4 BPA Exposure Decreases the Spine Density of Pyramidal Neurons in the CA1 Areas

**Figure 4:**
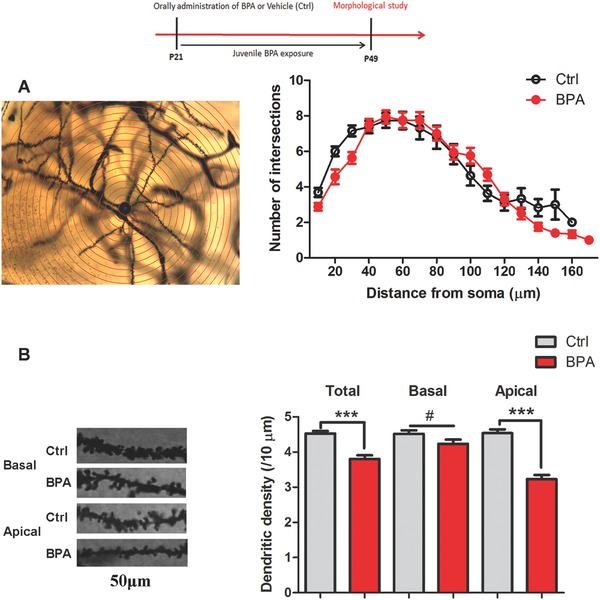
Alteration of dendritic morphology of pyramidal neurons in the hippocampal CA1 areas after juvenile BPA exposure. A) A representative Golgi‐Cox impregnated pyramidal neuron by Sholl analysis for measuring dendritic length. Sholl analysis and histograms plot showing no difference of dendritic length between the control and BPA exposed groups (*n* = 10 per group, *p* > 0.05); B) Representative sections (50 µm) of Golgi‐Cox stained apical and basal dendrites of pyramidal neurons in hippocampus. Histograms showing decreased dendritic spine density (spines per 10 µm), especially apical spine density, after juvenile BPA exposure. (*n* = 10 rats per group, ***p < 0.001).

Dendritic spines of pyramidal neurons are small protrusions from the dendritic shafts and receive most excitatory connections from presynaptic glutamatergic projections.^13^ A positive relationship between spine morphology and LTP induction has been demonstrated.^14^ Thus, we analyzed the length of dendritic shafts and spines density of pyramidal neurons in the hippocampal CA1 areas for exploring the morphological evidence for LTP impairment. A total of 10–12 pyramidal neurons in each control and BPA‐exposed rats were included for final analysis (*n* = 10 rats per group). All neurons were located in the dorsal part of the CA1 hippocampal region and Golgi‐Cox‐impregnated with apical and basal dendrites. According to Sholl analysis, total branch lengths in control and BPA‐exposed rats were 76.47 ± 5.24 and 76 ± 3.16 µm, respectively. There was no significant difference in intersection between dendrites and Sholl circles (dendritic arborization) in control and BPA‐exposed rats (*p* > 0.05, **Figure** **4**A), suggesting that the development of dendritic shafts was not affected by juvenile BPA exposure.

Spine density was analyzed with the MATLAB software as description in our previous paper.^39^ As shown in Figure 4B, there was a significant decrease in total spine density in BPA‐exposed rats, about 20% decrease in dendritic spine number compared with the control groups (*p* < 0.01, Figure 4B). These findings indicated that decreased spine protrusion resulted in reduced spine density, and BPA impaired spine formation in the hippocampus CA1 areas. Moreover, the densities of apical and basal spines also were quantified. We found that the apical spines density was decreased to two third of that of the control group (*p* < 0.01, Figure 4B). However, basal spines were not affected by BPA treatments (*p* > 0.05, Figure 4B). These results indicate that juvenile BPA exposure exerted a region specific effect on spine protrusion of dendritic shafts.

### 2.5 BPA Exposure Decreases the Glutamate Receptors Expression and Synaptic Transmission

**Figure 5:**
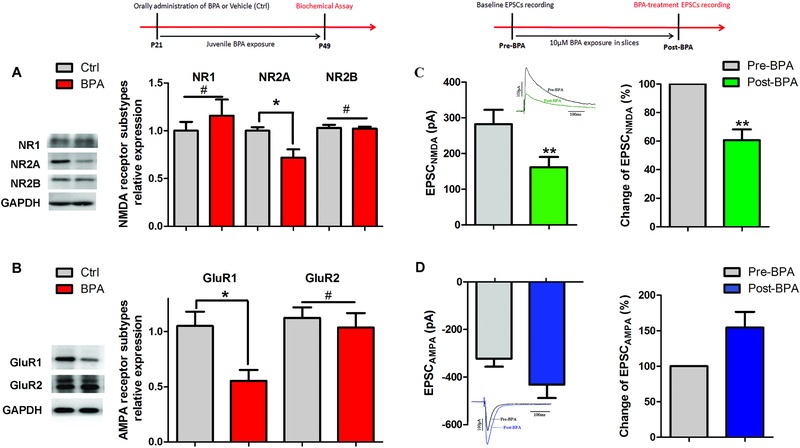
Downregulation of excitatory receptor and postsynaptic transmission after BPA exposure. A,B) Representative immunoblot and corresponding densitometric analysis showing the ratios of expression amounts of NMDA receptor subtypes (NR1, NR2A, and NR2B to GAPDH (#*p* > 0.05, *p < 0.05, and #*p* > 0.05, respectively) and AMPA receptor subtypes (GluR1 and GluR2) to GAPDH in control and juvenile BPA treated groups (*n* = 8 rats per group, *p < 0.05 and *p* > 0.05, respectively); C,D) Excitatory synaptic transmission in BPA‐treated brain slices (10 × 10^−6^ m) was assessed by recording NMDA and AMPA receptor mediated current in the hippocampal CA1 areas, as shown by decreased EPSC_NMDA_, not EPSC_AMPA_, after acute BPA treatment (*n* = 8 rats per group, *p < 0.05 and *p* > 0.05, respectively).

Induction of LTP at Sch‐CA1 synapses is dependent on glutamate receptors, especially NR2A, NR2B, GluR1, and GluR2.^15^ To further examine the synaptic mechanism behind LTP impairment, we assessed the effect of BPA on the expression of glutamate receptor subunits, including NR1, NR2A, NR2B, GluR1, and GluR2. As shown in **Figure** **5**A,B, there was a significant decrease in NR2A and GluR1 expression levels (*n* = 8 rats per group, *p* < 0.05) but not in the remaining subunits. These findings indicate that downregulation of postsynaptic glutamate receptors may decrease LTP after BPA exposure. Considering the expression alteration of NMDA and AMPA receptors, we investigated whether BPA impairs NMDA‐ and AMPA‐mediated synaptic transmission (*n* = 8 rats per group). By patch‐clamp recording, EPSC_NMDA_ and EPSC_AMPA_ were obtained from CA1 pyramidal neurons using bipolar tungsten‐stimulating electrodes with different holding voltages. As shown in Figure 5C, the EPSC_NMDA_ of synaptic responses was significantly decreased by about 40% after BPA treatment (10 × 10^−6^ m) compared with baseline values (282.13 ± 10.32 pA in pre‐BPA‐treated neurons, 151.88 ± 8.73 pA in post‐BPA‐treated neurons, *p* < 0.01). In contrast, BPA specifically led to a slight but nonsignificant increase of EPSC_AMPA_ (−310.94 ± 8.23 pA in pre‐BPA‐treated neurons; −644.59 ± 15.78 pA in post‐BPA‐treated neurons, *p* > 0.05, Figure 5D). These results suggest that synaptic transmissions mediated by NMDA receptors (NR) were deranged after BPA exposure, contributing to the reduced LTP.

### 2.6 BPA Exposure Reduces the Presynaptic Function as Well as Presynaptic Transmitter Synthesis and Release

**Figure 6:**
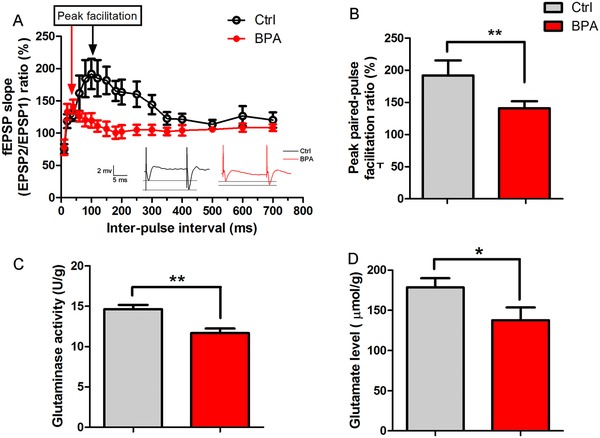
Decrease of pre‐synaptic transmission, and transmitter synthesis and release after juvenile BPA exposure. A,B) Paired‐pulse facilitation in the hippocampal CA1 areas was obviously inhibited in BPA exposed rats, as shown by reduced paired‐pulse ratio (fEPSP2/fEPSP1) (*n* = 10 rats per group, **p < 0.01); C,D) Glutamate synthesis was decreased by juvenile BPA exposure, as shown by downregulation of glutaminase activity and glutamate level (*n* = 8 rats per group, **p < 0.01, and *p < 0.05, respectively).

Paired‐pulse facilitation (PPF) is a presynaptic form of short‐term synaptic plasticity due to enhanced presynaptic transmitter release in response to the second of two closely spaced action potentials during field potential recording.^16^ Thus, we assessed whether altered presynaptic activity was also involved in LTP reduction after BPA exposure. The double pulse test with interpulse intervals (IPI) ranging from 10 to 700 ms was used to explore the change of PPF ratio (EPSP2/EPSP1 × 100, %). We found that decreased LTP amplitudes upon BPA application was accompanied by PPF inhibition (*n* = 10 rats per group, **Figure** **6**A). The PPF ratio at the peak of the synaptic enhancement was significantly decreased to 141 ± 11% compared to 191.83 ± 23% of control group (*p* < 0.01, *n* = 10 rats per group, Figure 6B). It suggests that BPA exposure could downregulate the presynaptic transmitter release in the hippocampal CA1 areas.

We also examined the glutamate synthesis potential and release after juvenile BPA exposure by ELISA methods to confirm PPF decrease. As shown in Figure 6C, glutaminase activity for glutamate synthesis was significantly inhibited after chronic BPA exposure (*n* = 8 rats per group, *p* < 0.05). Furthermore, glutamate release in BPA‐treated rats was decreased by 22.47% as compared to control (*n* = 8 rats per group, *p* < 0.05, Figure 6D). Decreased glutamate levels in the hippocampus also provide a likely explanation for presynaptic impairment of physiological function in BPA exposed rats.

## 3 Discussion

Combining behavioral assays and in vivo electrophysiology, the present study is the first to demonstrate that BPA exposure in juvenile rats deteriorated spatial memory and synaptic plasticity in the hippocampal CA1 areas. This involved the decrease of spine density on the dendrites of pyramidal neurons, especially the apical dendrite. Meanwhile, altered synaptic transmission also contributed to BPA‐induced cognitive impairment, with the decrease of presynaptic glutamate synthesis and release, and excitatory receptors expression.

Brain is one of the most sensitive body organs to environmental insults, especially in the developmental periods.^11^ In the present study, results of UPLC measurements demonstrate that there was a significant increase of BPA levels within the CSF after juvenile BPA exposure (Figure 1). This confirms that BPA could pass across the BBB,^17^ and thus exert toxic effect on the CNS. To assess the effect of juvenile BPA exposure on rat cognitive behaviors, two classic spatial memory test models were selected, including the MWM and Y‐maze tests, which are depended on the hippocampus to explore spatial environments.^18^ As shown in MWM training, BPA‐treated rats spent similar time for escaping compared to the control rats (Figure 2B), indicating no deterioration of memory acquisition ability. However, the hidden platform test reveals that BPA significantly decreased the memory retention for shorter platform duration in BPA‐exposed group than the control group (Figure 2C). Additionally, in the Y‐maze test (without stress punishment or reward), less time for exploring the novel arm was found in BPA‐treated group (Figure 2H). These results lend a strong proof that BPA exposure during the juvenile period perturbs hippocampus‐dependent spatial memory consolidation. Several investigations also have shown that prenatal and lactation BPA treatments result in impairment of hippocampal‐dependent spatial memory in MWM test in 3 weeks and 8 weeks old mice,^19^ 4 week old rats,[[qv: 7a]] and adult rats.^20^ Interestingly, those perinatal BPA exposure could cause the deterioration of spatial learning (memory acquisition) capability.^19^, ^20^ In the present study, BPA exposure in whole juvenile phase just has effect on the memory consolidation. It implies that the hippocampus in juvenile animals is less sensitive to BPA exposure than it in the first developmental stage, which is characterized with large amounts of mature synapses (a form of synaptic plasticity) until postnatal 21 d (end of lactation)^11^ and vulnerable to many chemical exposure, including nicotine, Pb, and polybrominated diphenyl ethers.^21^ However, BPA exposure from gestation to juvenile will have progressively worse effect on memory than perinatal exposure only. According to previous reports, one week BPA exposure (40 µg kg^−1^ d^−1^) in adolescent rats impairs the object placement performance, which is also hippocampal‐dependent spatial memory.^8^ Moreover, acute BPA exposure in adult rats could cause the impairment of object placement memory.^22^ Our findings provide important “missing” evidence of BPA effects on hippocampal‐related function in the whole life of rats. Taken together, behavioral results suggest that the juvenile stage before adolescence and adulthood is susceptible to BPA exposure, and exposure to BPA during this period can cause spatial memory deficits in the later life.

Synapses in the hippocampus constitute a major modulatory system that regulates normal learning and memory encoding processes, particularly spatial cognitive functions.^23^ For spatial memory impairment after juvenile BPA exposure, we hypothesized that there is an obvious reduction of hippocampal LTP within the synapses. Our results showed that juvenile BPA exposure inhibited LTP induction in the hippocampal CA1 areas after HFS of Sch in the CA3 areas (Figure 3B), which underlies BPA related memory deficits involving synapse plasticity changes. The “pathological plasticity” of synapse may provide the cellular basis of behavioral alterations. It has been found that 17β‐estradiol interaction with estrogen receptors has potential to heighten the magnitude of LTP at hippocampal CA3–CA1 synapses in rats.^24^ We speculate that BPA may exert inhibitory effects on the hippocampus‐related function in developmental rats by blocking estrogen receptors to disturb 17β‐estradiol effects in the normal physiological state.

Pyramidal neurons in the CA1 areas are a kind of spiny neurons, which have broadly distribution of dendritic spines on dendritic shafts to receive excitatory synaptic input.^13^ In the normal physiological situation, new spine could protrude from dendrites after LTP induction through F‐actin clustering and remodeling.^25^ Additionally, alterations of spine density are accompanied with functional changes at the synaptic level, including presynaptic input and synaptic strength.^14^ 17β‐estradiol has been shown to enhance dendritic apical spine density of CA1 pyramidal neurons in the hippocampus, via ERK and mTOR activation.[[qv: 24a,26]] Therefore, we explored whether juvenile BPA exposure alters spine morphology, which could help us understanding the effect of BPA on synaptic plasticity. Golgi‐cox staining results showed that four weeks BPA exposure (from 4 to 7 weeks old) did not change the dendritic branch length but obviously decreased the spine density of pyramidal neurons in the CA1 areas (Figure 4). This suggests that BPA can interfere with postsynaptic sites of excitatory synapses in pyramidal dendrites. It has been reported that four weeks BPA exposure in adult male vervet monkeys could decrease the number of excitatory synaptic inputs on dendritic spines of pyramidal neurons in hippocampus by electron microscopy analysis.[[qv: 5b]] In addition, prenatal BPA exposure results in reduced spine number on the hippocampal CA1 areas of neonates African green monkeys and adult mice (14 month old).[[qv: 9a,27]] Even one injection of BPA (40 µg kg^−1^) can reduce dendritic spine density in the CA1 areas of adult male rats.^22^ Although exposure time, BPA dosage and experimental subjects of our study are different with those previous studies, the results are consistent with their morphological alterations. Interestingly, BPA‐induced spine regressive alterations were restricted to the apical dendrite, not the basal dendrite (Figure 4B). According to 17β‐estradiol effects on apical spine,[[qv: 24a]] this finding further suggests that BPA‐induced structure and function deterioration are dependent on perturbing the functions of estrogen receptors on the hippocampus.

NR is considered one of the crucial synaptic elements for activity‐dependent synaptic plasticity. Over‐stimulating of NRs could increase Ca^2+^ influx, which then induces short‐ or long‐term alterations in the hippocampus, e.g., LTP.[[qv: 15a]] The different signaling properties of NRs are mainly due to their different subunit compositions.^28^ In the current study, decreased NR2A expression after juvenile BPA exposure (Figure 5A) is proposed to explain the postsynaptic mechanism of memory and synaptic plasticity impairment. In addition, the presence of GluR1 as well as the deficient of GluR2 in AMPA receptors has a contribution to LTP enhancement.[[qv: 15b]] In the hippocampus, the spine head size could be increased after LTP induction through AMPA receptor recruitment at synapses.^29^ Hence, the downregulation of AMPA receptors (GluR1) (Figure 5B) suggests that there is a lower regulative capability to control synaptic plasticity in the hippocampus after juvenile BPA exposure.^30^

Consistent with the result of NR expression, we also found significantly declined NMDA‐dependent current after acute BPA perfusion. To some extent, this provided the basal evidence for glutamatergic synaptic current in LTP and spatial memory deficits in juvenile BPA‐exposed rats. Interestingly, AMPA current had a nonsignificant increase in acute BPA‐treated slices, suggesting that there may be an obvious decline of EPSC_NMDA_/EPSC_AMPA_ ratio in BPA‐exposed rats. According to previous studies, 17β‐estrogen enhances learning and LTP magnitude by increasing the ratio of NMDA transmission to AMPA transmission,[[qv: 24c]] which underlies BPA exerting estrogen antagonist activity to induce reestablishment of excitatory synaptic transmission and impairment of synaptic plasticity.

PPF in the hippocampal CA1 areas is a transient form of presynaptic plasticity, which is considered an indicator of presynaptic transmitter release probability. It is well known that LTP expression includes the presynaptic locus associated with PPF ratio changes.^31^ In the present study, obviously decreased PPF ratio, glutamate release and glutaminase activity were observed after juvenile BPA exposure (Figure 6). These alterations provide the presynaptic mechanism of impaired LTP by BPA exposure. The above findings corroborate previous researches showing that perinatal BPA exposure results in less glutamate release from presynaptic neurons.^32^ Therefore, the presynaptic site in the hippocampus is susceptible to both perinatal and juvenile BPA exposure. In addition, developmental BPA exposure in cultured hypothalamic cells causes decrease of synapsin I phosphorylation,^33^ which can enable less vesicles to be released during the presynaptic depolarization.^34^ These findings indicate that BPA could also directly perturb the transmitter release from vesicles through regulating the activity of synapsins.

In conclusion, the present study shows that juvenile BPA exposure caused profound neurocognitive deterioration in spatial memory and synaptic plasticity. The decreased spine density, glutamate release, and NR2A and GluR1 expression may contribute to such functional deficits. Future studies are required to investigate the dose‐dependent effects of BPA on juvenile behaviors and explore the molecular mechanisms of BPA‐induced synaptic changes. The current findings shed light to understand the dynamics of BPA‐induced memory deterioration during the whole life.

## 4 Experimental Section

*Experimental animals and treatment*: Sprague–Dawley rats were obtained from Beijing Experimental Animal Center, P. R. China. All animal experiments were performed according to the National Institutes of Health Guide for the Care and Use of Laboratory Animals. The study was approved by the Institutional Animal Care and Use Committee of University of Science and Technology of China, P. R. China. Chronic BPA exposure was performed as previously described.^35^ Male rats were orally treated with or without BPA (1 mg kg^−1^ d^−1^, Sigma #239658, dissolved in dimethyl sulfoxide (DMSO)) from postnatal 21–24 to 49–52 d. In this study, one male rat pup per litter from different normal dams, mated with normal adult male SD rats, was used in the control or BPA‐exposed group (8–10 rats per group). The exposure concentration mentioned above was much lower than the currently accepted lowest observed adverse effect level (50 mg kg^−1^ d^−1^). In the control group, rats received equivalent DMSO volume instead of BPA (the highest DMSO volume was less than 0.5 mL).

*BPA detection*: SD rats with or without juvenile BPA exposure (*n* = 16 per group) were deeply anesthetized with urethane (10%, 1.8 g kg^−1^, intraperitoneal injection [IP]) and placed in the stereotactic frame. CSF samples (40–60 µL per rat) were then collected with 1 mL syringes from the third ventricle for analyzing CSF BPA concentrations. Sample preparation and BPA detection were carried out as described in previous studies.[[qv: 9a]] Briefly, CSF samples were diluted with 3 mL methyl tertiary butyl ether followed by twice extraction. The extract was evaporated to dryness under N_2_ (40 °C), and the residue reconstituted in 100 µL acetonitrile solution. Chromatographic separation for BPA was carried out on UPLC with fluorescence detection (Acquity UPLC BEH C18 1.7 µm, Waters, USA). The mobile phase (water and acetonitrile) was degassed in an ultrasonic bath before measurement. The column oven temperature was 40 °C, and the injection volume was 10 µL. Excitation wavelength was 227 nm and emission wavelength was 310 nm.

*Behavior tests: Morris water maze (MWM), Y‐Maze, and open field tests*: In this study, the effect of BPA on spatial learning and memory was evaluated in MWM and Y‐maze tests. Its effect on locomotion behavior was evaluated in the free exploration open field test.

*Morris water maze test*: The MWM maze test was performed as description in our previous studies with minor modification.^36^ Briefly, 49 d old SD rats with or without juvenile BPA exposure were subjected to the MWM test. The experimental device contained a circular tank (diameter: 1.5 m; depth: 0.5 m) with 0.25 m water (25 ± 1 °C), made opaque with black ink. Space learning was assessed over five consecutive days (training). Rats were allowed to swim to the hidden platform submerged 1.5 cm below the water surface for four trials every day (15 s per trial). The time spent for finding the hidden platform was recorded as the escape latency, which was inversely correlated with spatial learning ability. On the sixth day, platform locations were left entirety for spatial memory testing. Rats were submitted to a single search trial for 90 s (probe test). A starting position was randomly chosen in nonplatform quadrants and used for all animals. Escape latency and the duration in the platform location were recorded. Longer duration could reflect the spatial memory level. The total swimming distance and velocity in the water tank, represented as the basic motor function, were also recorded. All of parameters were estimated by EthoVision 8.5.

*Y‐Maze test*: The Y‐maze is based on the innate curiosity of rats to explore novel environment,^37^ and was further selected for assessing spatial memory without negative or positive reinforcers with or without BPA exposure. The Y‐maze device was made of black polyvinyl chloride (PVC), and consisted of three arms symmetrically separated by 120° (50 cm × 20 cm × 25 cm, length × width × height). In the present study, the three arms were randomly designated as home arm (always open), familiar arm (always open), and novel arm (block during training trial and open during test trial). During the training trial, rats were placed into the home arm of the maze and allowed to explore two arms (home and familiar arms) for 10 min. The maze was cleaned between each rat using 70% ethyl alcohol. After 2 d of training, rats were returned to the Y maze by placing them in the home arm on the third day (test trial). Then, the animals were allowed to explore freely all three arms of the Y maze for 5 min. The entry number and duration in each arm were recorded by video camera for each rat, and estimated by EthoVision 8.5. Under the same moving time on the Y‐maze, higher values of the two parameters in the novel arm reflected higher spatial memory ability.

*Open field test*: The open field test was selected to further assess locomotion (motor function) of rats with or without BPA exposure. The experiment was conducted in a free exploration open‐field apparatus, which was 100 cm × 100 cm × 50 cm (length × width × height) and divided into 4 × 4 squares. Each rat was carried into the open field and allowed to explore the apparatus (10 min daily; three consecutive days) for environment adaptation before testing. On the fourth day, rats were re‐exposed to the open field for 5 min tests. Performance (moving distance and velocity) of each rat also was recorded by video camera, and estimated by EthoVision 8.5.

*In Vivo field excitatory postsynaptic potential*: fEPSP was recorded to further evaluate the physiological function of the hippocampus after rats with or without BPA exposure, as described in our previous study.^38^ Briefly, 49–52 d old SD rats (*n* = 8 per group) were anesthetized with 20% ethylurethanm (1.5 g kg^−1^, IP) and positioned in a stereotaxic apparatus. Craniotomy was performed above the hippocampus corresponding to the representation of central vision. After surgical procedure completion, a tungsten bipolar stimulating electrode was placed into the Sch (Sch, which coordinates with the skull surface fat at Bregma: 4.2 mm posterior, 3.8 mm lateral, and 2.8 mm ventral). A glass recording electrode (3–5 µm tip diameter and 1–3 MΩ resistance) filled with 2 m NaCl was used to record fEPSP from the CA1 areas (Bregma: 3.4 mm posterior, 2.5 mm lateral, and 2.0 mm ventral). Before recording, a test pulse was performed to evoke the field potential and electrode position was readjusted until a maximal standard wave appeared. During the experiments, rectal temperature was monitored and maintained at 37 ± 0.5 °C by an automatic heating pad. Data were collected using Igor Pro 6.01 (Wave Metrics, OR, USA) and analyzed with Origin 8.0 software (Origin Lab, MA, USA).

*Input/Output functions*: To evaluate the effect of juvenile BPA exposure on basic synaptic transmission at the physiological level, the input–output (*I*/*O*) relationship was measured and shown as the *I*/*O* curve, the function of response (fEPSP) slope (mV ms^−1^) depended on the stimulus current (0.1–1.4 mA). Stimulus pulses were delivered at 0.05 Hz and three fEPSP slopes at each current were averaged. After *I*/*O* function recording was completed, the intensity of test stimuli (0.05 Hz) was adjusted to yield about 50% of the maximum response in the CA1 areas to induce paired pulse reaction (PPR) and LTP for evaluating the BPA effects on short‐term and long‐term synaptic plasticity.

*Paired‐Pulse response*: PPR was determined by delivering pairs of identical stimuli with IPI ranging from 10 to 700 ms to analyze the presynaptic transmitter release potential. Three responses were averaged at each IPI. The degree of PPR was quantified as paired pulse ratio, calculated as second peak response slope (second pulse; EPSP2)/first peak response slope (first pulse; EPSP1) (EPSP2/EPSP1). Three responses were averaged at each IPI.

*Long‐Term potentiation*: During LTP (physiological basis of synaptic plasticity) recording, a stable baseline for fEPSPs was first recorded for at least 20 min, and then LTP was elicited by applying HFS (five trains of 20 pulses at 200 Hz separated by 1 s, repeat six times at intervals of 1 min). Post‐tetanic recordings were performed for 1 h with single pulses at 0.05 Hz, and the response slopes in every 5 min were averaged and were normalized to baseline values.

*Golgi‐Cox staining and spine density assay*: After CSF collection, brain samples (with or without BPA exposure, *n* = 8 per group) were processed by the Golgi‐Cox staining method as we previously described.^39^ Briefly, brains were immerged in the Golgi‐Cox solution for 2 d (37 °C), and then were coronally sectioned into 200 µm thick slices by a vibratome (VT1000S, Leica, Germany). The sections containing the hippocampal CA1 areas were used in the present study. Those sections were then mounted to 2% gelatin‐coated slides and stained with ammonia for 60 min, followed by Kodak Film Fix for 20 min, and then dehydrated, cleared, and mounted using a resinous medium. Pyramidal neurons packed in the CA1 areas were imaged on a Nikon widefield microscope (Eclipse 80i, Japan) with a 20× objective; dendrite segments were imaged with a 100× objective. About 2–3 neurons per section were chosen and then 5–6 neurons from each brain were used for morphological analyses. Image J with Neuron J plugin^40^ and Sholl analysis were used for dendritic length analysis of pyramidal neurons. Additionally, the spine density (spine number per 10 µm dendrite) was calculated by using the MATLAB software.^39^ Three stretches of the secondary or third dendrite, each about 50 µm in length, were analyzed per neuron in.

*Detection of glutamate receptors expression by Western blot*: After CSF collection, brain samples (*n* = 8 per group) were removed from the skull quickly within 1 min and longitudinally cut into two halves; the hippocampus of right brain was used for protein extraction to assess the expression of glutamate receptors (NMDAR1/2A/2B and Glutamate receptor 1/2); the hippocampus of left brain was processed for glutamate transmitter release and synthesis. All the samples were stored at −80 °C until used for biochemical assays.

For Western blot, hippocampal samples were kept in ice‐cold lysis buffer and homogenized with a 1 mL syringe to extract tissue proteins. After 1 h incubation on ice, supernatants (containing hippocampal proteins) were collected by centrifugation at 4 °C (12 000 rpm, 10 min). Protein concentration was determined by the bicinchoninic acid (BCA) method. Equal amounts of samples were resolved by 8.5% or 4% sulphate‐polyacrylamide (SDS‐PAGE) gel. The resolved proteins were transferred to a polyvinylidene difluoride (PVDF) membrane and blocked with 5% bovine serum albumin and incubated with primary antibodies, glyceraldehyde‐3‐phosphate dehydrogenase (GAPDH, 1:10,000; catalog #Ab9484; Abcam), NMDAR1, NMDAR2A, NMDAR2B, glutamate receptor 1 (NR1, NR2A, NR2B, and GluR1; 1:1000; catalog #5704, #4205, #4207, and #13185, respectively; Cell Signaling Technology), and glutamate receptor 2 (GluR2, 1:1000; catalog #Ab20673; Abcam). Blots were then incubated with the appropriate horseradish peroxidase (HRP) conjugated anti‐mouse or anti‐rabbit secondary antibody (Cell Signaling Technology) and developed using enhanced chemiluminescence (GE Healthcare). Densitometry was assessed with the NIH Image J software. Protein expression was normalized to the GAPDH protein.

*Postsynaptic glutamate receptor‐Mediated currents recording*: To further analyze the effect of juvenile BPA exposure on postsynaptic‐glutamate‐receptor‐mediated synaptic transmission, patch‐clamp recording methods were used as described in our previous study.^38^ Briefly, young male SD rats (14–21 d old, *n* = 16) were decapitated, and the brains were quickly removed and submerged in cold (0–4 °C) artificial CSF (ACSF, in ×10^−3^ m: 124.00 NaCl, 2.69 KCl, 1.25 KH_2_ PO_4_, 2.00 Mg_2_SO_4_, 26.00 NaHCO_3_, 2.00 CaCl_2_, 10.00 glucose). Afterward, coronal hippocampal slices (300 µm thick) were cut with a vibrating microtome (VT‐1200S; Leica, Germany) and incubated in the oxygen saturated ACSF (>1 h, room temperature, 28 °C), which was continuously bubbled with a mixture of oxygen and carbon dioxide (95% O_2_ and 5% CO_2_). Then, the slices were transferred to a submerged recording chamber and then patch‐clamp recordings from somata of CA1 pyramidal neurons were performed in whole‐cell voltage clamp configurations using fire‐polished pipettes with a resistance of 3–5 MΩ.

To record glutamate currents, the ACSF was added with picrotoxin (50 × 10^−6^ m) to block GABA_A_‐mediated inhibition during the experiments. The NMDA receptor‐mediated postsynaptic current (EPSC_NMDA_) was then recorded at +40 mV with the addition of 6,7‐dinitroquinoxaline‐2,3‐dione (10 × 10^−6^ m) to block the AMPA component; the amplitude of this EPSC was measured at a delay of 100 ms; whereas AMPA receptor‐mediated current (EPSC_AMPA_) was recorded at −80 mV with the addition of (2R)‐amino‐5‐phosphonovaleric acid (AP5, 50 × 10^−6^ m) for blocking the NMDA component. The amplitude of this EPSC was measured with a delay of 20 ms. After acquisition of a stable baseline, BPA solution (10 × 10^−6^ m in ACSF) was normally administered 7–8 min for recording EPSC_NMDA_ and EPSC_AMPA_. Data were sampled using a computer equipped with the Clampex software (Version 10.30; Axon Instruments, America). Throughout this experiment, the liquid junction potential was not corrected, and the series resistance was periodically monitored but not compensated.

*Glutamate release and glutaminase activity assays*: To assess glutamate release in hippocampal tissues, assays were performed with the glutamate detecting kit (A074, Jiancheng, Nanjing, China). Briefly, hippocampal samples were homogenized with 1 mL syringe on the ice. Then, the homogenates were centrifuged at 2500 rpm for 10 min and the supernatant collections were used in the assay. According to the manufacturer's protocol of the glutamate assay kit, tissue extracts were mixed with different reagents. Finally, absorbance of the mixture at 340 nm was measured with a spectrophotometer at 37 °C (Elx800, BioTek, USA).

Glutaminase activity in hippocampal tissues was determined with an enzyme kit (A124, Jiancheng, Nanjing, China). Tissue specimens were homogenized on ice and centrifuged at 8000 rpm at 4 °C for 10 min. The supernatants were mixed with different reagents and centrifuged at 8000 rpm for 10 min at room temperature. Finally, the supernatants were added with the last reaction mix, and absorbance of the mixture was measured at 420 nm.

*Statistical analysis*: All of data were expressed as mean ± S.E.M. Unpaired two‐tailed t‐test and repeated measures analysis of variance (ANOVA) were used to evaluate statistical differences between control and BPA‐exposed groups. Statistical analysis was conducted using the GraphPad Prism 5.0 software and *p* < 0.05 was considered as the statistical difference.

## Supporting information

Supplementary
