# Supplementary material for: Bisphenol A Impairs Synaptic Plasticity by Both Pre‐ and Postsynaptic Mechanisms
Source: Adv Sci (Weinh). 2017 Apr 19;4(8):1600493. doi: 10.1002/advs.201600493 (PMC5566242; doi:10.1002/advs.201600493)
Supplement: Supplementary file 1 — Supplementary [file ADVS-4-na-s001.pdf]

Copyright WILEY-VCH Verlag GmbH & Co. KGaA, 69469 Weinheim, Germany, 2017.

ADVANCED SCIENCE
Open Access

Supporting Information

for *Adv. Sci.,* DOI: 10.1002/advs.201600493

Bisphenol A Impairs Synaptic Plasticity by Both Pre- and Postsynaptic Mechanisms

Fan Hu, Tingting Li, Huarui Gong, Zhi Chen, Yan Jin, Guangwei Xu, and Ming Wang**

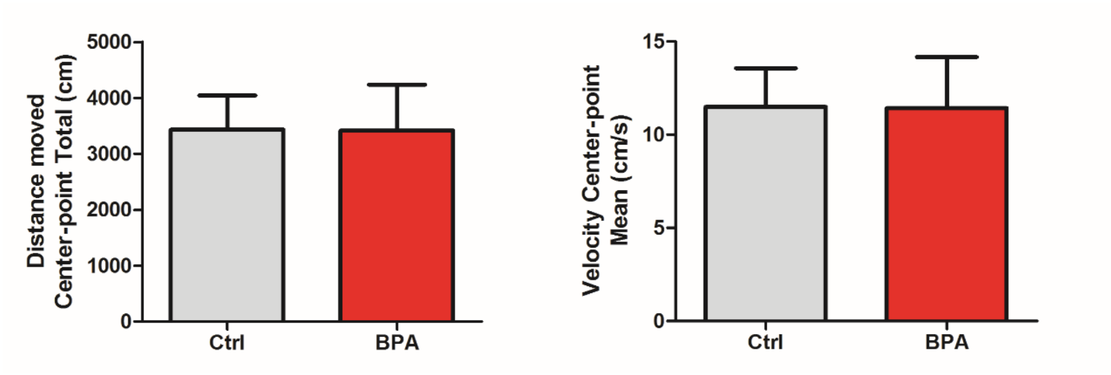

Juvenile BPA exposure had no effect on the locomotion (moving distance and velocity) of rats in open field test.
